# Navigating motherhood: biological and landscape factors affecting postpartum movement in white-tailed deer

**DOI:** 10.1186/s40462-024-00517-2

**Published:** 2024-12-18

**Authors:** Angela M. Holland, Jacob M. Haus, Justin R. Dion, Joseph E. Rogerson, Jacob L. Bowman

**Affiliations:** 1 Department of Entomology and Wildlife Ecology, University of Delaware, 531 S College Avenue, Newark, DE 19716 USA; 2 Department of Biology, Bemidji State University, 1500 Birchmont Drive NW, Bemidji, MN 56601 USA; 3 Wildlife Division, Oregon Department of Fish and Wildlife, Salem, OR 97302 USA; 4 Delaware Division of Fish and Wildlife, 6180 Hay Point Landing Road, Smyrna, DE 19977 USA

**Keywords:** Cervid, Displacement, Fawn, Female, *Odocoileus virginianus*, Parturition, Space use

## Abstract

**Background:**

Population growth and management in cervid species is dependent on reproductive ecology and factors influencing juvenile survival. Aspects of the female’s movement behavior likely affect juvenile survival and movement patterns of pregnant and lactating females differ from non-pregnant or non-lactating females. Explanations for these differing movement patterns include change in nutritional demands for the female, isolation during parturition, and predator avoidance. White-tailed deer (*Odocoileus virginianus*) are an important managed cervid and a better understanding of their reproductive ecology, including the relationships between resources, movement, and juvenile survival, can better inform management.

**Methods:**

Our objective was to determine if biological factors, such as female age, fawn age, number of fawns, as well as characteristics of prepartum range affected the female’s postpartum daily movement or overlap of space used pre- and postpartum in Sussex County, Delaware, USA (2,420 km^2^). We collected GPS locations 2 weeks pre- and postpartum on 22 individual females from 2016 to 2017. In total, we recorded data from 263 days of postpartum movement for an average of 12 days/individual. We used a hierarchical modeling process to test biological factors and prepartum home range characteristics on two aspects of postpartum movement behavior, mean hourly displacements and daily use of prepartum home range.

**Results:**

Mean hourly displacement decreased with increased female age and increased with number of known fawns alive and the female’s home range size prior to parturition. We found that as fawns aged the doe increased use of the prepartum home range.

**Conclusions:**

Our results indicate that younger females are moving more than older females during lactation potentially to access higher quality habitat. This increased movement increases nutritional demand and may play a role in fawn survival. Females are more likely to use more of their prepartum home range as fawns age, a finding congruent with previous research. This differentiation in metric response (movement rate vs. space use) emphasizes the complexities of movement ecology and the importance of considering multiple dependent variables for complex behavior.

## Background

Reproductive ecology and factors influencing juvenile survival can have implications for population growth and management in cervid species. To understand these limitations, research on juvenile survival often focuses on the condition of the juvenile [e.g., sex, birth mass, 1–3] and the female [e.g., age, mass, parity status, 3–6]. However, aspects of the female’s movement behavior likely affect juvenile survival. Movement patterns of pregnant and lactating females differ from non-pregnant or non-lactating females (i.e., never pregnant or early juvenile loss) in cervids with juveniles following the “hider” and “follower” strategies [7–9]. Explanations for these differing movement patterns include change in nutritional demands for the female, isolation during parturition, and predator avoidance [10–12].

Gestation and lactation are two of the most nutritionally demanding aspects of a mammal’s life history. This phenomenon is observed in white-tailed deer (*Odocoileus virginianus*) with increased energy costs during the final trimester of gestation and lactation; however, the period of peak milk yield (10–27 days postpartum) is the most energetically costly [13]. Increases in crude protein requirements during late gestation and lactation are 50–100% greater than requirements for routine maintenance for females [14]. The increased energetic expenditure during peak lactation cannot be offset by nutrient intake, and females are operating at a metabolic deficit during this time [13].

Availability of resources needed for increased nutritional demands during lactation could be dependent on quality of forage within the female’s home range. Due to social status, older females maintain relatively stable home ranges throughout late pregnancy and lactation and push away younger females with home ranges overlapping their core areas [15–17]. Although dominance status is more likely a function of body mass rather than age [18, 19], body size increases with age until females are approximately 4 years old [20]. By reducing range size and isolating from other individuals, older females have exclusive access to known and potentially better-quality resources within their home range [21, 22]. Younger females, however, are forced to find alternative and possibly suboptimal resources, which results in differences in spatial patterns of younger and older females during late pregnancy and lactation [17].

Ultimately, fawns of young females forced out of the range of an older female may experience reduced survival. Young females may not find sufficient resources in their new range, or they may have to travel greater distances between adequate food resources and suitable cover for their fawns. A lack of resources or the need to increase travel time, and therefore energetic costs and time away from their fawns, reduces nutrition availability for the fawns and may reduce survival. Previous studies found reduced fawn survival for younger females [3, 5, 22].

Our objective was to determine if biological factors such as female age, fawn age, number of fawns, as well as characteristics of prepartum range affected the female’s postpartum daily movement or overlap of space used pre- and postpartum. We hypothesized younger females were excluded from high quality foraging areas (estimated with landcover metrics) resulting in increased rates of postpartum movement and decreased use of their prepartum space. We also hypothesized that movement and space use were affected by the number and age of living fawns. We predicted that the rate of postpartum daily movement would be negatively correlated with fawn age but positively correlated with the number of fawns, while the degree of prepartum space used would be positively correlated with fawn age but negatively correlated with the number of fawns.

## Methods

### Study area

**Fig. 1:**
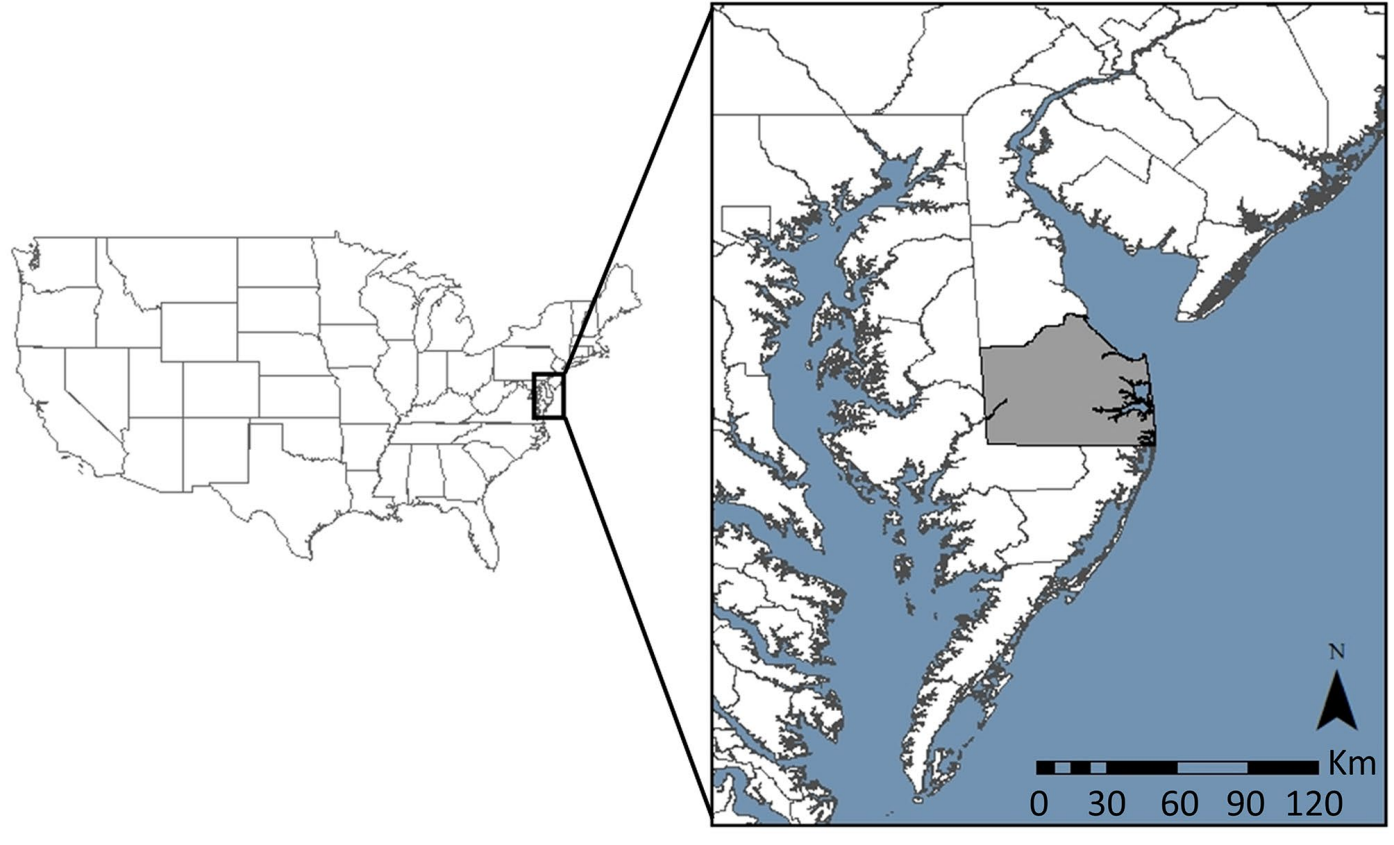
We monitored the postpartum movements of female white-tailed deer throughout Sussex County (gray), Delaware, USA during spring (April–June) of 2016–2017

We conducted our research in Sussex County, Delaware, USA (2,420 km^2^; Fig. 1), which consisted of a mixed forest-agricultural landscape. The county was comprised of the following land cover types: agriculture (42%), forest (including woody wetland; 35%), development (14%), open water or herbaceous wetland (8%), and less than 1% each of bare rock, shrub, and grassland [23]. Major agricultural crops were corn, soybeans, and winter wheat [24]. The topography of Sussex County was flat with elevation ranging from 0 to 21 m [25]. The deer density in Sussex County was 19 deer/km^2^ [aerial survey, 26] and the mean parturition date was 28 May [3], with peak parturition timed to follow spring green-up. Although the carnivore species typically associated with predation on white-tailed deer were rare within the study area, parturient females still demonstrated anti-predator strategies in their parturition behaviors [27].

Temperatures during the study (2016–2017) ranged from − 14 °C to 36 °C. Average annual precipitation during the study was 122 cm; comparable to the 30-year (1981–2010) average of 119 cm [28]. Spring and summer precipitation averaged 78 cm, with temperatures ranging from 2 °C to 36 °C [29].

### Capture and monitoring

We captured female white-tailed deer using rocket nets, drop nets, and Clover traps baited with whole kernel corn from December–April of 2015–2017 [30]. We captured, immobilized, and handled animals following procedures outlined in Haus et al. [31]. We aged deer using tooth replacement and wear [32] and fit all females ≥ 1.5 years of age with radio collars and vaginal implant transmitters from Advanced Telemetry Systems (Isanti, Minnesota, USA). We deployed GPS collars (model G2110E2 [820 g]) with vaginal implant transmitters (model M3930U [23 g]) linked to the collar via ultra-high frequency radiotelemetry [rVIT, 33] on 44 females. We followed established guidelines for rVIT deployment [34, 35] and inserted rVITs to a depth of 20 cm. We monitored animal condition and vital signs (temperature, heart rate, respiration) until individuals left the capture site under their own power. We received remote notifications for GPS collared females including fix locations and rVIT status daily until 1 May. Following 1 May, we received GPS locations and rVIT status reports every hour. GPS collars recorded hourly location data from 1 May–30 June in 2016 and 1 May–15 June in 2017.

We captured and monitored fawns for mortality [for more details, see 3]. We located individuals every 12 h during their first 28 days of life to ensure timely notification of mortality status [36, 37]. The University of Delaware Institutional Animal Care and Use Committee approved all capture and handling procedures (protocol #1288).

### Variable calculation and data analysis

We subset all GPS data by removing any physically impossible locations (e.g., locations in the ocean) but did not remove locations based on dilution of precision error screening [38]. Landscape characteristics associated with location bias such as rugged topography and heavy canopy cover [39–41] are not typical within the study area. Only one female was recaptured with successful location of fawns in 2016 and 2017, and we considered this individual with multiple years of location data as separate individuals for each year because behavior changes as deer age [42].

We estimated prepartum occurrence distributions (hereafter, prepartum range) using location data for the 15 days prior to, but not including the parturition date (e.g., parturition on 16 May, prepartum range estimated for 1–15 May). We calculated prepartum ranges for each female using the dynamic Brownian Bridge Movement Model in the ‘move’ package [43] in program R [44]. We informed the model with a window size of 7, margin of 3, and location error of 10 m. Window and margin values specified the length of a moving window in which values of motion variance were estimated based on change points in the animal’s movement path. Allowing motion variances to reflect changes in animal movement behavior provided more accurate utilization distributions relative to traditional Brownian Bridge Movement Models [45]. The earliest parturition date was 9 May, so all individuals had at least 8 days of hourly locations plus prior daily location data to establish their prepartum range (Table 1).

**Table 1:** Location and movement data summary by white-tailed deer females from Sussex County, Delaware, USA, 2016–2017. Table has a row of data for each female per year with female age (Age), parturition date (Date), number of fawns collared (# Fawns), data from prepartum points (Prepartum) including number of points in analysis (# Points), mean hourly displacement in meters averaged across days with hourly data (Displacement), size of prepartum range in hectares (Range), Shannon diversity index of prepartum range (SDI), mean shape index of prepartum range (MSI), data from postpartum points (Postpartum) including number of days in analysis (Days), number of points in analysis (# Points), mean hourly displacement in meters (Displacement), and average daily percent of postpartum points in the prepartum range (% Range). For each individual Displacement and % Range is the average of available days. Overall means across individuals are presented for # Points, Prepartum Range, SDI, and MSI. Mean, minimum and maximum values for all individual-days are presented for pre- and postpartum Displacement and % Range

Age	Date	# Fawns	Prepartum					Postpartum			
			# Points	Displacement	Range	SDI	MSI	Days	# Points	Displacement	% Range
4	5/24/2016	2	306	107.9	61.2	1.04	1.78	9	181	94.3	87.5
4	5/9/2016	1	194	77.6	19.3	0.73	1.18	13	257	71.6	41.9
3	5/20/2016	1	331	110.3	61.3	0.93	1.54	11	218	111.2	94.1
3	5/23/2016	1	322	68.4	17.8	0.71	1.38	12	236	48.8	81.3
5	5/22/2016	2	315	65.2	18.8	1.00	1.65	14	275	45.2	96.4
5	5/31/2016	2	330	64.2	15.3	0.65	1.39	13	256	49.1	89.4
6	6/1/2017	2	335	52.9	12.6	0.69	1.40	13	310	63.8	77.1
4	5/26/2016	2	309	86.6	37.2	0.92	1.57	11	218	82.1	93.0
3	6/1/2016	1	322	65.0	33.3	0.32	1.44	13	297	57.0	78.5
3	5/27/2016	2	320	71.8	26.0	0.58	1.37	13	256	29.5	96.0
3	5/20/2016	2	313	61.8	23.9	0.34	1.63	6	122	66.4	95.9
3	6/2/2017	2	367	118.0	45.3	1.03	1.70	12	288	101.8	34.4
2	5/15/2017	2	313	94.2	43.5	0.77	1.47	14	332	101.3	99.4
4	5/22/2017	2	335	75.2	30.7	1.14	1.53	14	330	78.9	95.6
4	5/19/2017	2	336	114.3	66.5	0.62	1.56	14	317	117.5	0.0
6	6/4/2017	2	337	69.2	38.5	0.62	1.41	11	264	68.2	60.2
3	5/28/2017	2	342	89.1	52.2	0.88	1.80	14	332	86.0	98.4
2	5/28/2017	2	335	101.0	35.8	0.57	1.45	14	331	116.9	9.6
5	5/30/2017	2	336	73.0	23.2	0.82	1.41	14	332	86.5	91.3
4	6/10/2017	2	335	108.7	42.5	0.61	1.40	4	94	122.8	97.9
2	6/4/2017	1	353	82.2	30.6	0.50	1.34	10	239	121.6	92.5
4	5/23/2017	2	335	84.3	40.5	0.57	1.63	14	333	148.8	50.0
Mean			324	81.6	35.3	0.73	1.50	12	264	84.0	73.53
Minimum Daily Value				11.5						13.4	0.0
Maximum Daily Value				301.2						255.1	100.0

To improve accuracy of the land cover uses, we merged the land cover layer from the 2016 National Land Cover Database [23] with a Wetland Reserve Program property layer from the U.S. Department of Agriculture Natural Resource Conservation Service in ArcMap 10.7.1 (ESRI, Redlands, California, USA) [46]. We then reclassified the land cover into open water, developed, grassland, forest (including woody wetland), shrub, agriculture (including pasture and row crop), emergent wetland, and Wetland Reserve Program. For all additional spatial analyses, we used program R, including the ‘sf”, ‘raster’, and ‘landscapemetrics’ packages [47–49]. We clipped each prepartum range to the land cover raster and calculated class and landscape metrics for each female each year. Metrics of interest included the landscape shape index, Shannon diversity index, mean shape index, contagion, and size of prepartum range (ha). These variables have previously been associated with hypotheses of resource availability for white-tailed deer [50].

To address our objective, we measured daily movement and continued use of the prepartum range for 14 days postpartum. For each female for each day after parturition (e.g., parturition on 16 May, daily estimates for 17–30 May), we calculated the average distance moved each hour by measuring the distance between subsequent GPS locations and dividing by the time between locations to account for missing hourly data, hereafter referred to as mean hourly displacement. We also counted the number of postpartum location points inside and outside the prepartum range to calculate the percentage of points in the prepartum range, hereafter percent prepartum-range use. We removed any postpartum day that did not have at least 19 data points for the 24-hour period (> 75%). This removed individuals from the dataset that did not give birth during the hourly data collection window (i.e., after 30 June 2016 or 15 June 2017). Additionally, we determined the number of surviving collared fawns for every female each day, the age of the fawns (days since parturition if living), and categorized female age as mature (≥ 4 years old) or immature (< 4 years old) [3].

We tested for collinearity between variables using a Pearson correlation test and excluded 1 or both variables from the same model if |r| > 0.6. Prepartum range size was correlated with landscape shape index, and Shannon diversity index was correlated with contagion. We removed landscape shape index and contagion from further analyses because prepartum range size and Shannon diversity index were more easily interpreted. As expected, the number of surviving fawns was correlated with fawn age, so these variables were not used in the same models. Female age and female maturity were also not used in the same models because we created female maturity using female age, thus, these variables were highly correlated.

We assessed which variables affected daily female movement by fitting linear, mixed-effects models in a maximum likelihood framework to assess mean hourly displacement with individual female ID as the random effect. To assess daily postpartum use of the prepartum range, we fit logistic regression models with mixed-effects to assess the probability postpartum points fell within the prepartum range by using the ‘cbind’ function with the number of points inside and outside of the prepartum range and individual female ID as the random effect. We conducted analyses for both response variables in program R using the ‘lme4’ package [51].

We created a priori model sets for each response variable (i.e., mean hourly displacement and percent prepartum-range use). We approached modeling with a two-step process, first we identified which biological variables best predicted each response variable and then used the variable(s) from the top biological model in combination with the landscape variables to create a landscape variable model set. This approach reduced the total number of models fit by eliminating additional combinations of biological and landscape variables. We fit biological variables first because there is a larger body of research supporting the effect of these variables on female postpartum movement and allowed us to account for this known variation before addressing variation due to aspects of the landscape associated with resource availability. The model set for biological variables included 8 models. We used female age, female maturity, number of fawns, and fawn age (as an interaction of days since parturition and a binary indicator of whether at least one fawn was alive) each in a model and then combinations of female age or female maturity with number of fawns or fawn age. The second model set focused on landscape variables and had 7 models. Each model included the variable(s) from the top biological model and univariate or all possible combinations of landscape variables: prepartum range size, Shannon diversity index, and mean shape index.

We used Akaike’s Information Criterion scores corrected for small sample sizes (AICc) to determine the top model in both model sets for each dependent variable [52]. Due to the hierarchical nature of the analysis, we only used variables from the top biological model in the landscape model set. We considered models within 2 ΔAICc of the top model to be competing within the landscape model set [52].

## Results

Of the 44 captured individuals, we identified and collared at least 1 fawn for 26 parturition events due to a ~ 40% failure rate for either rVITs or GPS collars [33]. An additional 4 females were removed from the study when they did not meet the daily 19-locations requirement for any day postpartum. We used 22 individuals in our analysis with an average female age of 4 years. In total, we recorded data from 263 days of postpartum movement for an average of 12 days/individual (Table 1). Hourly mean displacement was slightly more variable during prepartum period than postpartum period (Table 1).

**Fig. 2:**
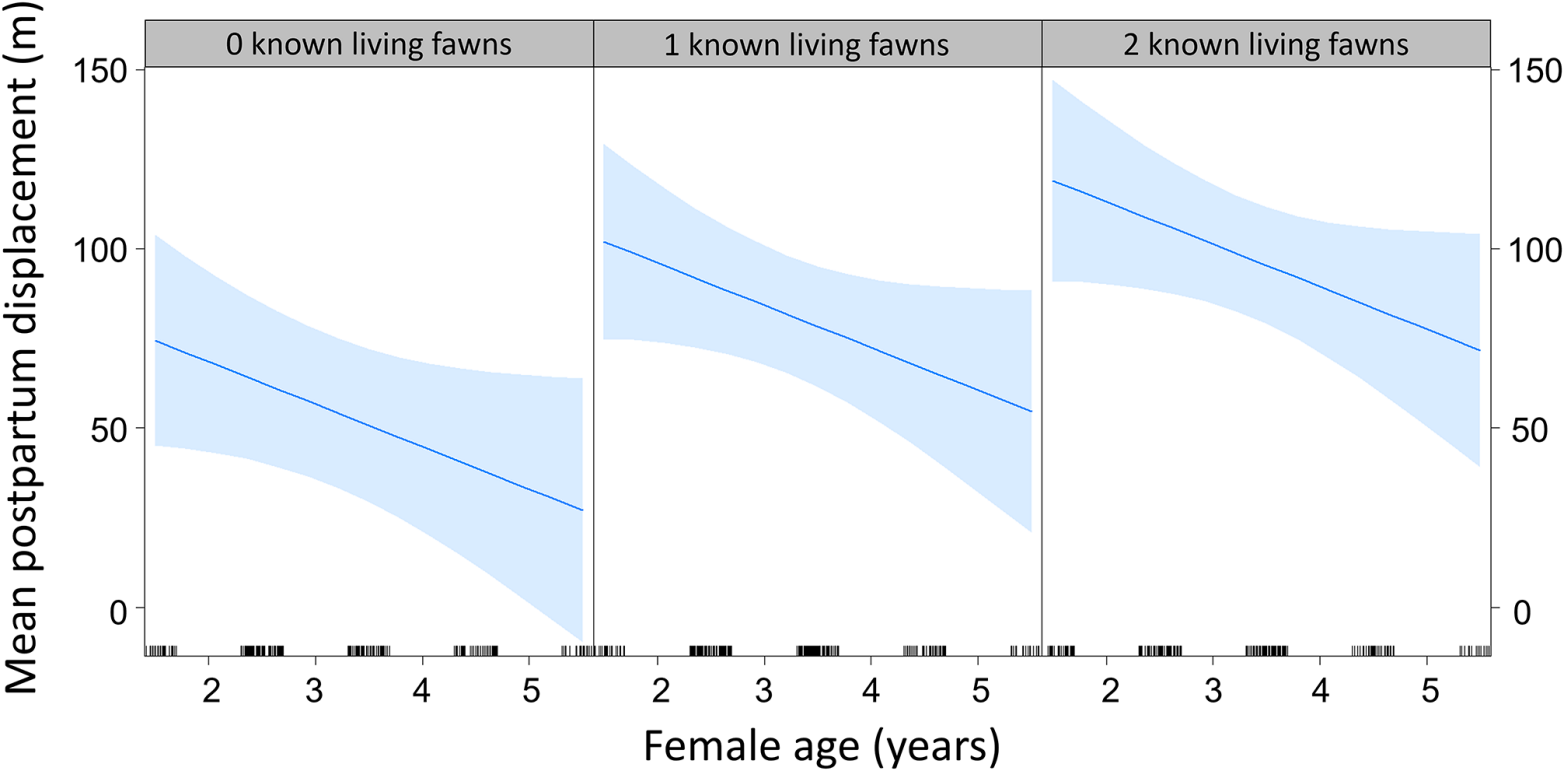
Model output from the top overall model for mean hourly displacement based on age of white-tailed deer female and number of known living fawns in Sussex County, Delaware, USA, 2016–2017

The top model in the biological model set for mean hourly displacement included female age and the number of fawns living. The second model only included number of fawns living (ΔAICc = 1.25; Table 2). We used female age and number of fawns living as the base model for the landscape model set. The top model in the landscape model set included size of the prepartum range in addition to the biological variables (Table 2). After parturition, older age females had shorter mean displacements than younger age females (β = -7.17, SE = 5.66) and increased with both number of fawns (β = 19.16, SE = 4.39) and size of the prepartum range (β = 1.14, SE = 0.42; Fig. 2). The second model (ΔAICc = 1.45) included the biological variables, prepartum range, and mean shape index, however the standard error for mean shape index exceeded the parameter estimate and this variable was not considered informative. The top 4 models included the biological variables and prepartum range size (cumulative AICc weight = 0.89).

**Table 2:** Model results for biological and landscape model sets for mean hourly displacement (Dis.) of postpartum females in Sussex County, Delaware, USA, 2016–2017. Models within each set are ranked based on the lowest Akaike’s Information Criterion adjusted for small sample size (AICc) where ΔAICc = AICc_*i*_ – minimum AICc, *K* = number of parameters, *w* = AICc weight, and *LL* = log likelihood

	Model	ΔAICc	K	w	LL
Biological	Dis. ~ Female Age + Number of Fawns	0.00	5	0.56	-1305.61
	Dis. ~ Number of Fawns	1.25	4	0.30	-1307.27
	Dis. ~ Female Maturity + Number of Fawns	3.30	5	0.11	-1307.26
	Dis. ~ Female Age + Fawn Age^a^	7.48	7	0.01	-1307.25
	Dis. ~ Fawn Age	7.70	6	0.00	-1308.41
	Dis. ~ Female Maturity + Fawn Age	9.81	7	0.00	-1308.41
	Dis. ~ Female Age	16.74	4	0.00	-1315.02
	Dis. ~ Null	16.87	3	0.00	-1316.12
	Dis. ~ Female Maturity	18.93	4	0.00	-1316.11
Landscape	Dis. ~ Female Age + Number of Fawns + Prepartum Range Size	0.00	6	0.44	-1302.51
	Dis. ~ Female Age + Number of Fawns + Prepartum Range Size + Mean Shape Index	1.45	7	0.21	-1302.17
	Dis. ~ Female Age + Number of Fawns + Prepartum Range Size + Diversity Index	2.01	7	0.16	-1302.46
	Dis. ~ Female Age + Number of Fawns + Prepartum Range Size + Mean Shape Index + Diversity Index	3.58	8	0.07	-1302.17
	Dis. ~ Female Age + Number of Fawns	4.11	5	0.06	-1305.61
	Dis. ~ Female Age + Number of Fawns + Mean Shape Index	5.68	6	0.03	-1305.34
	Dis. ~ Female Age + Number of Fawns + Diversity Index	6.00	6	0.02	-1305.50
	Dis. ~ Female Age + Number of Fawns + Diversity Index + Mean Shape Index	7.77	7	0.01	-1305.33
	Dis. ~ Null	20.99	3	0.00	-1316.12

^a^Fawn Age includes days since parturition, fawn survival status, and an interaction between these variables

The top model in the biological model set for proportion of points in the prepartum range was the model depicting fawn age as an interaction between days since parturition and fawn survival status. The second model included fawn age and female maturity (ΔAICc = 1.29; Table 3) with the model including fawn age and female age following close behind (ΔAICc = 1.75). We used the biological variable fawn age as the base model for the landscape model set because, while the models including female maturity and age were competitive, the standard error was larger than the parameter estimates in both models and these variables were not considered informative. No landscape models ranked better than the top biological model (Table 3) and additional variables in all competing models had larger standard error values than parameter estimates. As the age of living fawns increased the proportion of postpartum points in the prepartum range also increased (days since parturition: β = 0.03, SE = 0.03; fawn survival status: β = 0.41, SE = 0.31; days since parturition × fawn survival status: β = 0.09, SE = 0.03).

**Table 3:** Model results for biological and landscape model sets for percentage of postpartum female locations in the prepartum ranges (%R) in Sussex County, Delaware, USA, 2016–2017. Models within each set are ranked based on the lowest Akaike’s Information Criterion adjusted for small sample size (AICc) where ΔAICc = AICc_*i*_ – minimum AICc, *K* = number of parameters, *w* = AICc weight, and *LL* = log likelihood

	Model	ΔAICc	K	w	LL
Biological	%R ~ Fawn Age^a^	0.00	5	0.51	-823.32
	%R ~ Female Maturity + Fawn Age	1.29	6	0.27	-822.91
	%R ~ Female Age + Fawn Age	1.75	6	0.21	-823.14
	%R ~ Number of Fawns	83.65	3	0.00	-867.21
	%R ~ Female Maturity + Number of Fawns	84.91	4	0.00	-866.81
	%R ~ Female Age + Number of Fawns	85.44	4	0.00	-864.08
	%R ~ Null	86.12	2	0.00	-869.47
	%R ~ Female Maturity	87.45	3	0.00	-869.11
	%R ~ Female Age	87.99	3	0.00	-869.38
Landscape	%R ~ Fawn Age	0.00	5	0.27	-823.32
	%R ~ Fawn Age + Diversity Index	1.20	6	0.15	-822.87
	%R ~ Fawn Age + Prepartum Range Size	1.22	6	0.15	-822.88
	%R ~ Fawn Age + Prepartum Range Size + Mean Shape Index	1.76	7	0.11	-822.09
	%R ~ Fawn Age + Prepartum Range Size + Diversity Index	1.81	7	0.11	-822.12
	%R ~ Fawn Age + Mean Shape Index	1.87	6	0.11	-823.20
	%R ~ Fawn Age + Prepartum Range Size + Mean Shape Index + Diversity Index	3.17	8	0.06	-821.73
	%R ~ Fawn Age + Diversity Index + Mean Shape Index	3.31	7	0.05	-822.87
	%R ~ Null	86.12	2	0.00	-869.47

^a^Fawn Age includes days since parturition, fawn survival status, and an interaction between these variables

## Discussion

Our results indicate that older females have higher movement efficiency but does not fully support our hypothesis that younger females are excluded from high quality feeding areas since age did not affect changes in space use. Smaller prepartum range also decreased postpartum displacement indicating that individuals with access to areas with higher quality forage and cover had lower energetic demands. Additionally, we found support for our hypothesis that postpartum daily movement and space use is related to the number and age of living fawns. Postpartum daily movement was positively related to the number of living fawns, and prepartum space use during postpartum was positively related to fawn age. We did not find support for our prediction that postpartum daily movement would be affected by fawn age or a relationship of prepartum space use during postpartum with number of fawns. This differentiation in metric response (movement rate vs. space use) to the biological variables emphasizes the complexities of movement ecology.

Mean hourly displacement was positively correlated with both the number of surviving fawns and prepartum range size which supports an association between postpartum displacement and energetic demand. Home range size is often used as an indicator of the nutritional quality within the range, such that individuals occupying poorer quality areas must have larger ranges to meet their nutritional demands [53–55]. Similarly, an increase in the number of nursing fawns would inherently increase the female’s metabolic deficiency.

Previous research has demonstrated even minor reductions in maternal nutrient intake during lactation can affect fawn behavior and survival. For instance, Therrien et al. [56] found a 20% reduction from an *ad libitum* food supply for lactating females lead to reduced rates of growth and survival for their fawns. The greater postpartum displacements we observed in younger females suggests they must expend more energy to either find available food resources or to avoid aggressive interactions with more socially dominant females. Additionally, young females are still allocating energy to their individual growth, which increases their nutritional demands [20]. Such an increase in the metabolic deficit for young females is likely associated with the reduced rates of fawn survival for these individuals. In the absence of carnivore species typically associated with fawn predation, Dion et al. [3] observed reduced survival rates of fawns born to immature females (< 4 years old) relative to mature females (≥ 4 years old). In that study, emaciation was the most common condition related to fawn mortality; however, mean birth masses were similar between fawns born to immature and mature females. Similar birth masses suggest the nutritional deficiencies contributing to reduced fawn survival in immature females were occurring during the postpartum period [3]. When considering the results of our study, the nutritional deficiencies leading to the emaciation of fawns were likely due to increased movement rates of the female, which reduced time available to forage and increased energy expenditure.

Contrary to our hypothesis that older females would exclude younger females from their prepartum range during their postpartum movements, we did not observe an effect of female age or maturity status on the percentage of daily postpartum location points that occurred in the prepartum range. A critical assumption of our initial hypothesis was the prepartum ranges, which were likely shared by female social groups, are also the best fawning territories. While prepartum areas occupied by matriarchal female groups may provide quality nutrition, females regularly shift their space use around the time of parturition [9, 12, 57, 58], suggesting a trade-off between areas of nutritional quality and areas of greater cover for fawns or the need to increase fawn spacing for predator avoidance. Due to this shift in resource use, the percentage of daily postpartum location fixes within the prepartum range does not likely reflect the social hierarchy within matriarchal groups. We did, however, observe females progressively increasing use of the prepartum range during the postpartum period, and use of the prepartum range was greater for females with living fawns than for females who had lost their fawns. If the prepartum range is an area of quality nutritional resources for the female, the increasingly frequent use of this area during the postpartum period likely relates to increases in both the mobility of the fawn as well as an increasing metabolic deficit during lactation [13, 59]. Females who lost their fawns would experience a reduction in their metabolic deficit, and aggression from females with fawns may limit return to their prepartum range.

**Fig. 3:**
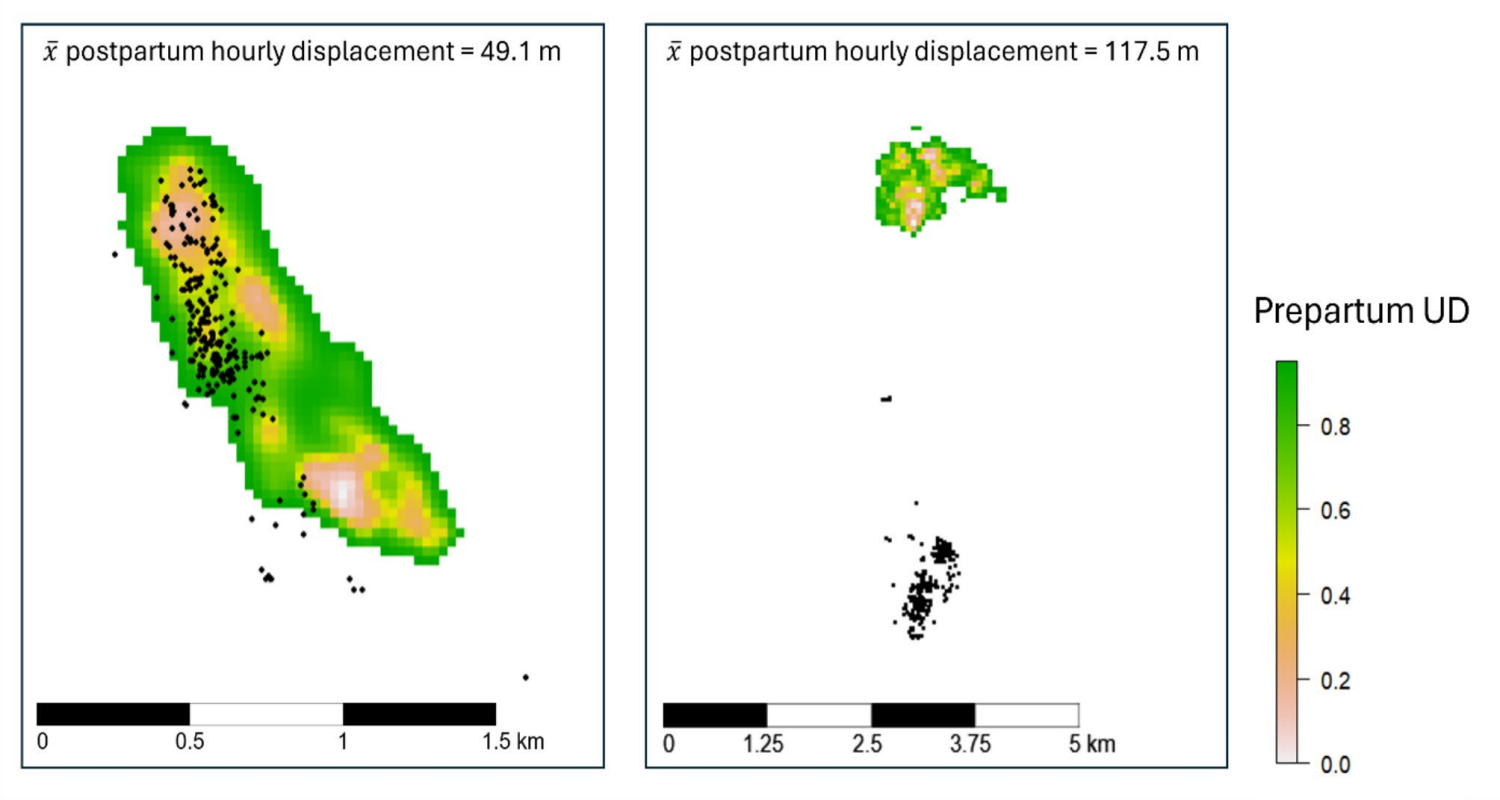
Examples of 95% utilization distributions (UD) from 2 parturient white-tailed deer (15–0 days prepartum) in southern Delaware, USA overlayed with postpartum location fixes (hourly points for 14 days after parturition). The 5-year-old female on the left had 2 fawns, one died after 4 days and one survived beyond the postpartum study period, 89.4% of postpartum location fixes occurred within the prepartum UD. The 4-year-old female on the right had 2 fawns, one died after 11 days and one survived beyond the postpartum study period, 0% of postpartum locations occurred within the prepartum UD

Our sample size for number of individual females was relatively small and variation in individual behavior may have affected our ability to identify overall trends (Fig. 3). For example, one female moved 6.5 km between pre- and postpartum ranges resulting in 0% of points in the prepartum range for every postpartum day, and another moved ranges (1-km shift) after the death of her 2-day old fawns (Table 1). This unique behavior likely affected our ability to determine relationships between pre- and postpartum range use with our biological and landscape variables. Similarly, when identifying parturition events from movement data, variation in individual’s movement patterns reduced the ability to accurately identify the parturition window for white-tailed deer, however the same approach worked well for elk (*Cervus canadensis*) [9].

In conjunction with our hypothesis that postpartum movement ecology is driven by resource requirements of the female is the hypothesis that fawn spacing for predator avoidance drives female movement ecology [60]. These hypotheses are not mutually exclusive and may work in tandem to determine female movement. Under a predator avoidance hypothesis, during the cryptic phase fawns should be spaced apart to prevent a predator from finding multiple neonates [60]. Competition for fawning space may force some females to move farther from their pre-partum range than others due to age, social status, or timing of parturition [5, 58]. Our study found support for the effect of anti-predator strategies on female movement due to increased fawn numbers increasing the daily movement rate of females. Spacing of multiple neonates required additional movement for the female to care for both individuals in separately located bedding sites. Although our study area did not have any established predator populations at the time of the study, previous work in the area indicates that anti-predator behaviors persist in the population [27].

Complexity in female movement ecology is due to multiple drivers affecting behavior. Nutritional demands, necessity of anti-predator behavior, and parturition timing varies between individuals and over time for an individual. These changes may cause issues in establishing patterns of postpartum behavior [58], and in our study, this is the likely cause for differences in ability to predict our dependent variables. Our dependent variables were selected to focus both on the location of female movement (postpartum space use) as well as the amount of movement (daily movement rate). Differences in predictions for these aspects of movement ecology for both our biological and landscape variables emphasizes the complexity of movement ecology and the importance of considering multiple dependent variables for complex behavior. Ultimately, we found that over time females move their fawns closer to their prepartum range, increasing use, but still have increased movement rates based on the number of fawns living. Movement rates are additionally dependent on the size of the prepartum range and the age of the female.

Our results suggest differences in postpartum movement behaviors reflect a gradient in metabolic deficiency for younger and older females in white-tailed deer, which likely has fitness implication in the form of reduced fawn survival [i.e., 3]. Management actions attempting to offset fawn mortality rates via predator removal or habitat improvements associated with fawning cover will not likely achieve the desired demographic response unless females of all ages have access to quality nutrition during lactation.

## Data Availability

The dataset supporting the conclusions of this article is property of the State of Delaware and cannot be shared openly due to state policy. Interested parties can contact the corresponding author to establish data sharing.
